# Ciliary Ift20 and Wwtr1 Coordinate Transforming Growth Factor‐β Receptor II Signaling to Maintain Bone–Fat Balance in Skeletal Homeostasis

**DOI:** 10.1002/mco2.70916

**Published:** 2026-09-20

**Authors:** Yang Li, Yang Yang, Shuting Yang, Huijuan Chang, Yi‐Ping Li, Shuying Yang

**Affiliations:** ^1^ Department of Basic and Translational Sciences School of Dental Medicine University of Pennsylvania Philadelphia Pennsylvania USA; ^2^ Department of Psychiatry and Behavioral Sciences School of Medicine Johns Hopkins University Baltimore Maryland USA; ^3^ Division in Cellular and Molecular Medicine Department of Pathology and Laboratory Medicine Tulane University New Orleans Louisiana USA; ^4^ Center for Innovation & Precision Dentistry School of Dental Medicine University of Pennsylvania Philadelphia Pennsylvania USA; ^5^ The Penn Center for Musculoskeletal Disorders School of Medicine University of Pennsylvania Philadelphia Pennsylvania USA

**Keywords:** bone homeostasis, Ift20, osteoblasts, TβRII, Wwtr1

## Abstract

The balance between bone formation and bone marrow adipose tissue accumulation is essential for skeletal integrity, and disruption of this balance contributes to skeletal degeneration, osteoporosis, and metabolic bone loss. However, the molecular mechanisms controlling this balance across skeletal homeostasis and disease remain unclear. Here, transcriptomic analysis of human osteoporotic bone samples revealed significant downregulation of Ift20 and Wwtr1 in osteoblast‐lineage cells. Using osteoblast‐specific knockout models, we found that loss of both Ift20 and Wwtr1 in the osteoblast lineage caused severe bone loss and excessive bone marrow adipose tissue accumulation, which was further aggravated under ovariectomy and high‐fat diet conditions. Mechanistically, Ift20 localized to primary cilia and interacted with transforming growth factor‐β receptor II (TβRII), protecting it from c‐Cbl‐mediated ubiquitination and degradation to sustain TGF‐β signaling. Meanwhile, Wwtr1 directly bound the *TβRII* promoter to promote its transcription. Loss of Ift20 and Wwtr1 converged on TβRII suppression, impairing osteoblast differentiation while enhancing adipogenesis and osteoclastogenesis. These findings reveal a previously unrecognized cilia–transcription–TGF‐β regulatory mechanism that governs osteoblast–adipocyte fate decisions and skeletal remodeling. The Ift20–Wwtr1–TβRII axis represents a critical safeguard of bone mass and marrow fat balance and may provide a therapeutic target for osteoporosis and metabolic bone diseases.

## Graphical abstract

Deletion of Ift20 and Wwtr1 in osteoprecursors promoted adipogenic differentiation while suppressing osteogenesis and bone remodeling through dysregulation of TGF‐β signaling. Mechanistically, Ift20 stabilized TβRII by inhibiting c‐Cbl‐mediated ubiquitination and proteasomal degradation. In parallel, Wwtr1 functioned as a transcriptional regulator that directly controlled endogenous TβRII expression.

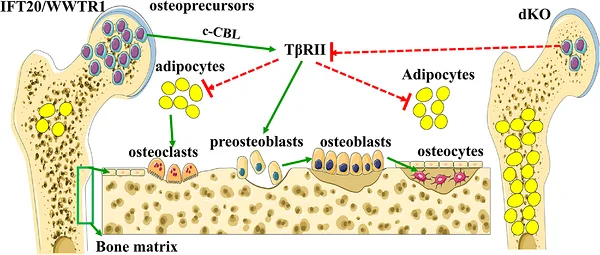

## 1 Introduction

Disorders characterized by impaired bone–fat balance, including osteoporosis (OP), represent major causes of skeletal fragility and fracture. OP is a progressive skeletal disease marked by reduced bone mass, deterioration of bone microarchitecture, and increased fracture risk [1, 2]. Affecting millions globally, OP carries an enormous economic burden, with direct healthcare costs in the United States alone exceeding $17.5 billion annually [3]. A common pathological feature of OP and other metabolic bone disorders is the expansion of bone marrow adipose tissue (BMAT) [4, 5, 6], which frequently expands at the expense of bone formation [7, 8, 9]. This inverse relationship reflects an imbalance in the lineage commitment of bone marrow mesenchymal stem cells (BMSC), favoring adipogenic differentiation at the expense of osteogenic differentiation [10, 11]. Despite its clinical importance of this bone–fat imbalance, the molecular mechanisms that coordinate osteoblast differentiation and marrow adiposity under pathological conditions remain poorly understood.

Bone marrow represents a unique microenvironment in which osteoblasts and adipocytes coexist and share progenitors [12]. Osteoblast dysfunction characterized by suppressed differentiation and decreased expression of key transcription factors such as runt‐related transcription factor 2 (Runx2) and osterix (OSX) [13, 14] is a major contributor to osteoporotic bone loss [15, 16]. Concurrently, BMAT accumulation, observed in both aging and high‐fat diet (HFD)‐induced bone loss models, further impairs the bone‐forming niche, and exacerbates skeletal deterioration. Thus, identifying regulators that coordinate this reciprocal relationship between osteogenesis and adipogenesis is critical for developing effective therapeutic strategies to restore bone–fat balance.

Intraflagellar transport 20 (Ift20) is a ciliary trafficking protein that plays a vital role in skeletal development and osteoblast function [17, 18]. Our previous studies demonstrated that Ift20 promotes osteogenesis while suppressing adipogenesis by maintaining primary cilia‐dependent signaling in mesenchymal stem cells. Specifically, loss of Ift20 skews BMSC lineage commitment toward adipocytes at the expense of osteoblast differentiation. In addition, osteoblast‐specific deletion of Ift20 disrupts bone development by impairing osteoblast alignment and polarity, further compromising bone formation [19, 20]. WW domain‐containing transcription regulator 1 (Wwtr1, also known as TAZ), a central effector of the Hippo signaling pathway, is likewise a critical determinant of bone–fat lineage allocation [21, 22, 23, 24]. Wwtr1 promotes osteoblast differentiation by co‐activating Runx2‐driven transcription and concurrently represses adipogenic programs through inhibition of peroxisome proliferator‐activated receptor gamma (PPARγ) activity, thereby favoring osteogenesis over adipogenesis [22, 25, 26]. Genetic loss of Wwtr1 in mice leads to reduced bone mass and increased marrow adiposity [27], highlighting its essential role in maintaining skeletal integrity and restraining fat accumulation within the bone marrow niche. Notably, global deletion of Wwtr1 also results in shortened primary cilia in cystic epithelia, accompanied by downregulation of ciliary proteins such as kinesin family member 3A (Kif3a) and polycystin‐1 (Pkd1) [28, 29], suggesting a functional intersection between Wwtr1 signaling and ciliary regulation. Consistently, combined heterozygous loss of Pkd1 and Wwtr1 produces additive defects in osteogenesis and enhances adipogenesis compared with single deficiencies of Pkd1 or Wwtr1 [25]. Importantly, both Ift20 and Wwtr1 have been identified as pathogenic drivers of cystic kidney disease in clinical settings [27, 29], implying a shared or convergent regulatory mechanism. Through integrative transcriptomic analysis of human osteoporotic bone samples [30], we identified marked downregulation of both Ift20 and Wwtr1 in osteoblast‐lineage cells. Together, these findings support a model in which Ift20 and Wwtr1 cooperatively regulate bone–fat balance by integrating cilia‐dependent signaling with transcriptional control of lineage commitment. However, the extent to which these factors functionally interact and the molecular mechanisms underlying their coordination in bone homeostasis remain largely unexplored.

In this study, we identify a previously unrecognized Ift20/Wwtr1–TβRII regulatory axis, that is, critical for maintaining bone homeostasis by suppressing marrow adiposity. Using osteoblast‐specific knockout models, we demonstrate that combined loss of Ift20 and Wwtr1 results in severe bone loss and marrow adipose tissue accumulation, which are further exacerbated by ovariectomy (OVX) and HFD. Mechanistically, Ift20 stabilizes TβRII at the ciliary base by preventing c‐Cbl‐mediated ubiquitination and proteasomal degradation, whereas Wwtr1 directly enhances TβRII transcription. These findings reveal a dual regulatory mechanism through which ciliary trafficking and transcriptional control converge on TGF‐β signaling to safeguard bone–fat balance, and they highlight Ift20 and Wwtr1 as potential therapeutic targets for OP.

## 2 Results

### 2.1 Ift20 and Wwtr1 Signatures Are Decreased in Osteoblast‐Lineage Cells Derived From Human and Mouse Osteoporotic Bone

**FIGURE 1:**
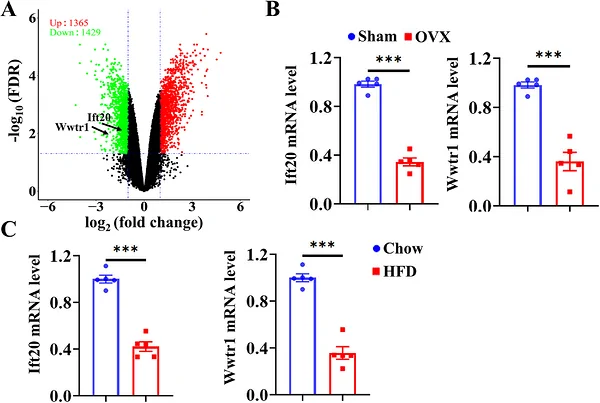
Ift20 and Wwtr1 signatures are decreased in human and mouse samples with osteoporosis. (A) A Volcano plot of transcriptome profiles in osteoblast‐lineage cells from the patients (females) with osteoporosis (GSE35958). *n* = 5. (B and C) Relative mRNA level of *Ift20* and *Wwtr1* in primary osteoblasts from the calvaria of the OVX‐ (B) or HFD‐induced (C) osteoporotic mice. *n* = 5. Error bars: SEM. ^***^ *p* < 0.001.

To investigate the potential roles of Ift20 and Wwtr1 in bone homeostasis and osteoporotic bone loss, we first analyzed their expression using publicly available transcriptomic data from patients with OP (GSE35958) [30]. Our data revealed that Ift20 and Wwtr1 expressions are significantly decreased in osteoblast‐lineage cells from the bone of osteoporotic patients compared to the controls (Figure 1A). To further assess the relevance of these findings in experimental models of pathological bone loss, we examined Ift20 and Wwtr1 expression in OVX‐ and HFD‐induced osteoporotic mouse models. Consistent with human data, expression of Ift20 and Wwtr1 was markedly decreased in primary calvarial osteoblasts isolated from mice 6 weeks after OVX (Figure 1B), as well as in osteoblasts from HFD‐induced osteoporotic mice (Figure 1C), compared with respective control groups. Together, these results indicate that Ift20 and Wwtr1 are consistently downregulated in osteoblast‐lineage cells under osteoporotic conditions and suggest that they may serve as critical regulators of bone homeostasis and OP pathogenesis.

### 2.2 Double Deletions of Ift20 and Wwtr1 in Osteoblasts Cause a Severe OP‐Like Phenotype

**FIGURE 2:**
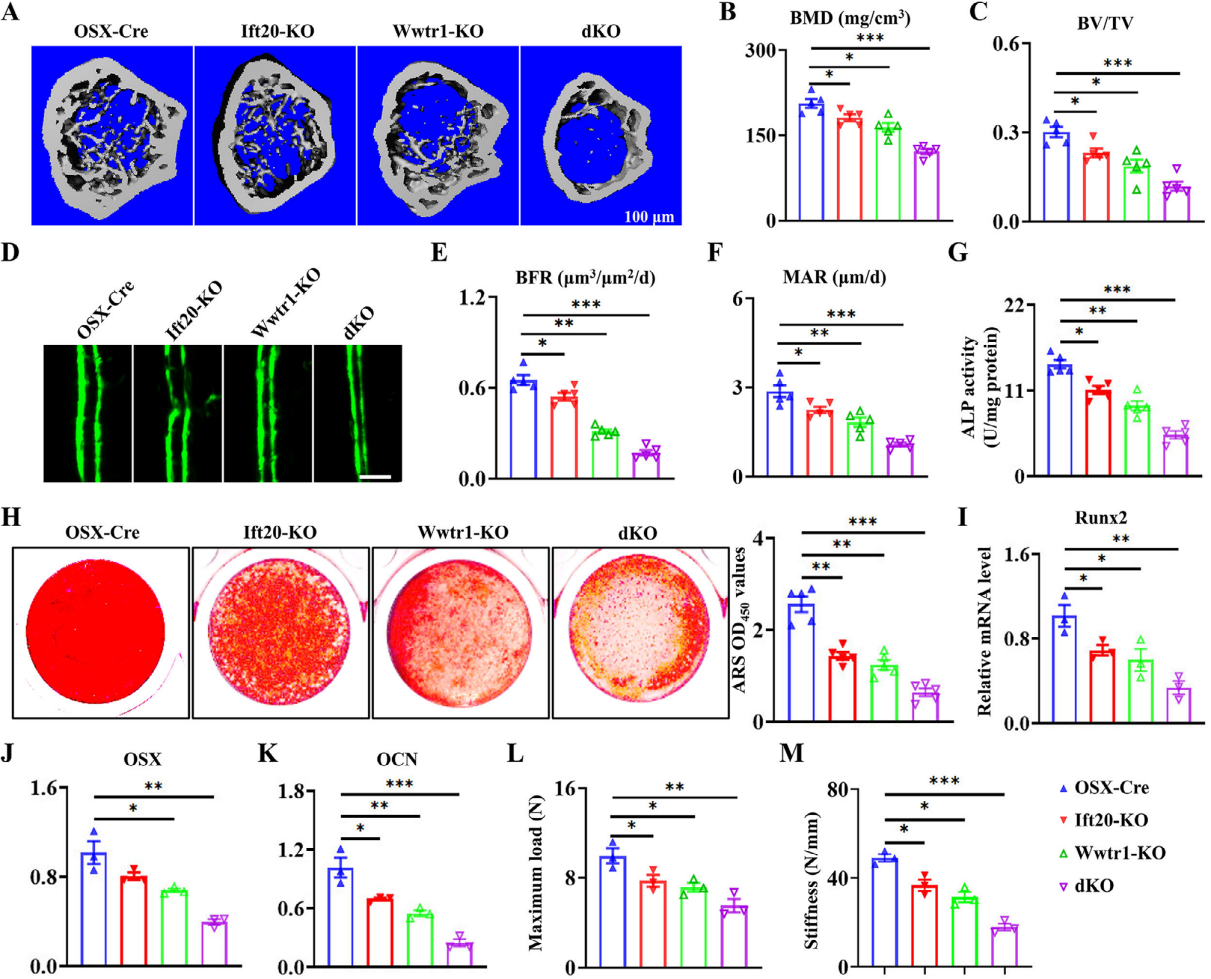
Double deletions of Ift20 and Wwtr1 in osteoprogenitor cells cause a severe osteoporosis‐like phenotype. (A) Representative µCT images of femurs from 3‐month‐old Ift20‐KO, Wwtr1‐KO, dKO mice, and age‐matched controls. Scale bars, 100 µm. *n* = 5. (B and C) Quantitative measurements of BMD and BV/TV of distal femurs (A). *n* = 5. (D) Representative images of calcein double labeling of proximal tibias from 3‐month‐old Ift20‐KO, Wwtr1‐KO, dKO mice, and age‐matched controls. Scale bar, 20 µm. *n* = 5. (E and F) Quantification of bone formation rate (BFR) and mineral apposition rate (MAR) from (D) as indicated. *n* = 5. (G) ALP activity in primary osteoblasts after stimulation with osteogenic media for 5 days as indicated. *n* = 5. (H) Representative images of ARS staining of primary osteoblasts after stimulation with osteogenic media for 2 weeks as indicated. The corresponding quantitative analysis of ARS staining is based on (H) at right. *n* = 5. (I–K) Analysis of osteogenic markers by qRT‐PCR after stimulation with osteogenic media for 2 weeks as indicated. *n* = 3. (L and M) Quantitative analysis of bone strength of femurs at 3 months by maximum load (L) and bone stiffness (M) as indicated. *n* = 3–5. Error bars: SEM. ^*^ *p* < 0.05, ^**^ *p* < 0.01, ^***^ *p* < 0.001.

To investigate the function of Ift20 and Wwtr1 in bone formation and homeostasis, we generated three gene conditional knockout lines with single and double deletions of Ift20 and Wwtr1 in osteoblasts by breeding Ift20 and Wwtr1 single and double‐floxed lines with OSX‐Cre transgenic mice (hereafter named Ift20‐KO, Wwtr1‐KO, and double‐knockout [dKO]). RT‐PCR verified that their expressions were efficiently extinguished in primary osteoblasts (Figure S1A,B). Intriguingly, we found that double deletions of Ift20 and Wwtr1 in osteoblasts resulted in additive bone loss and displayed a severe OP‐like phenotype compared to the single deletion of Ift20 or Wwtr1 in osteoblasts (Figure 2A), as evidenced by 41%, 12.04%, and 20.3% reduction in the bone mineral density (BMD) in Ift20‐KO and Wwtr1‐KO, dKO mice compared to the controls (OSX‐Cre mice) (Figure 2B). Consistently, we found the femurs from dKO mice lost approximately 50% of bone volume per total volume (BV/TV) (Figure 2C). Histomorphometry analysis of femur metaphysis showed that loss of Ift20 and Wwtr1 in osteoblasts resulted in significant bone loss in the trabecular bones compared to the age‐matched controls (Figure 2D–F). To further identify the function of Ift20 and Wwtr1 in osteogenesis, we isolated primary osteoblasts from calvaria of Ift20 and Wwtr1 double and single deficient mice and controls to characterize their osteogenic potential in vitro. As expected, we found that the alkaline phosphatase (ALP) activity was significantly decreased in dKO mice after stimulation with osteogenic induction media for 5 days (Figure 2G). Consistently, the mineralized nodule formation (alizarin red S [ARS]) also displayed a significant decrease in dKO mice after stimulation with osteogenic induction media for 2 weeks (Figure 2H). Moreover, we found that deletions of Ift20 and Wwtr1 in primary osteoblasts significantly decreased the expression of osteogenic markers such as Runx2, OSX, and osteocalcin (OCN) (Figure 2I–K). We also tested the strength of the femurs isolated from 3‐month‐old Ift20‐KO, Wwtr1‐KO, dKO mice, and age‐matched controls (OSX‐Cre) with a three‐point bending assay. As expected, we found that the bone strength and stiffness were significantly reduced in dKO mice compared to the single‐knockout mice and controls (Figure 2L,M).

### 2.3 Ift20 and Wwtr1 Are Required for Maintaining Bone Homeostasis in Osteoblasts

**FIGURE 3:**
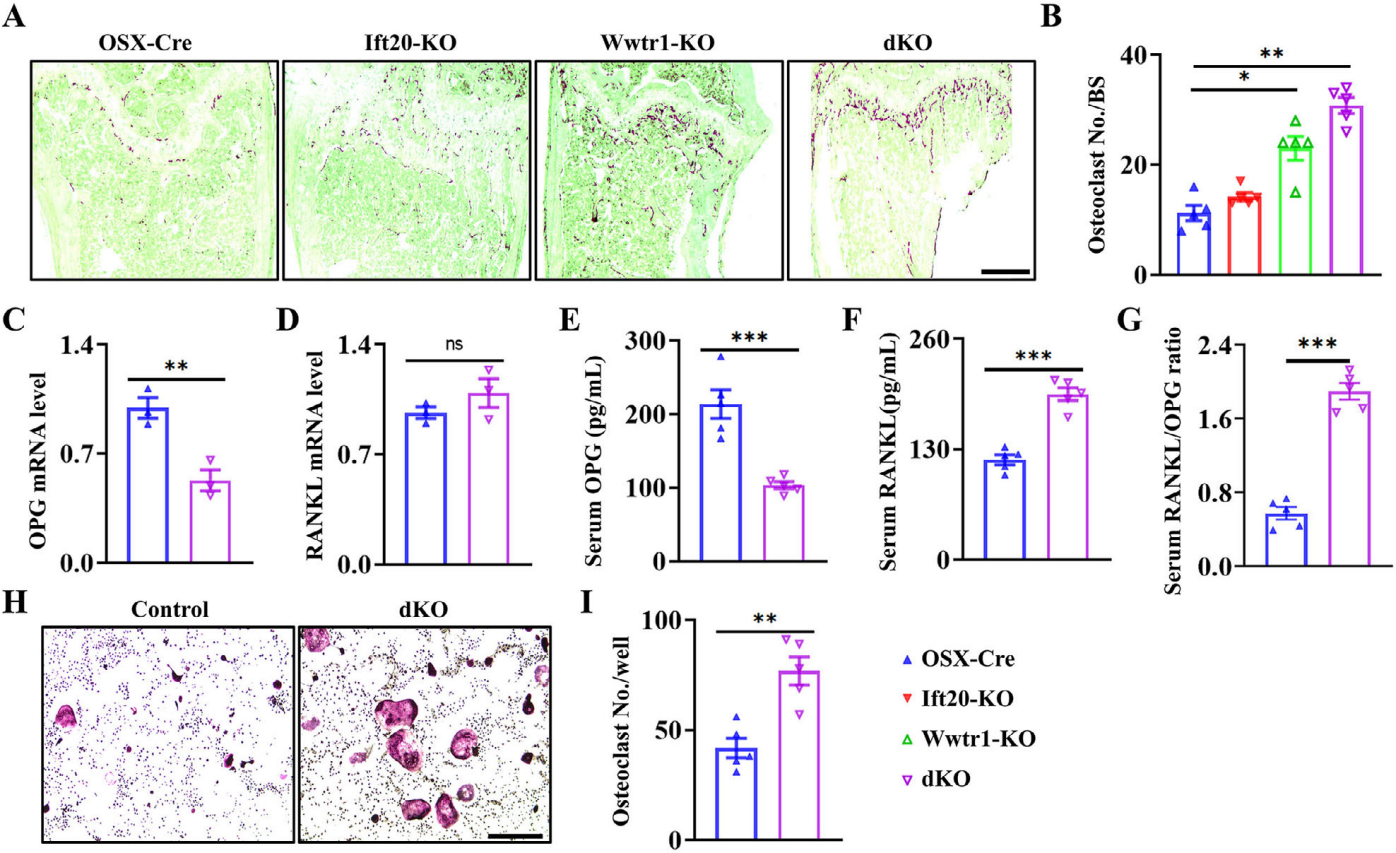
Ift20 and Wwtr1 deficiency in osteoprogenitor cells augments osteoclastogenesis and reduces bone remodeling. (A) Representative TRAP‐stained images of femurs from 3‐month‐old Ift20‐KO, Wwtr1‐KO, dKO mice, and controls (OSX‐Cre). Scale bar, 200 µm. (B). Quantitative analysis of osteoclast numbers per bone surface in A. *n* = 5. (C and D) qRT‐PCR analysis of OPG and RANKL in primary osteoblasts from dKO mice and age‐matched controls. *n* = 5. (E–G) The serum levels of OPG and RANKL and serum RANKL/OPG ratio from 3‐month‐old dKO mice and age‐matched controls. *n* = 5. (H and I) TRAP staining of osteoblastosteoclast co 10;cultures from 3‐month‐old dKO and control mice for 7 days (H). Scale bars, 100 µm. Quantitative analysis of TRAP staining in (I). *n* = 5. Error bars: SEM. ^*^ *p* < 0.05, ^**^ *p* < 0.01, ^***^ *p* < 0.001. ns, no significant difference.

Bone formation is a dynamic process, that is, tightly regulated and maintained by osteoblast‐mediated bone formation and osteoclast‐mediated bone resorption [31, 32, 33, 34]. The results from tartrate‐resistant acid phosphatase (TRAP) staining showed that TRAP^+^ osteoclasts were significantly enhanced in the dKO mice compared to the single Ift20 or Wwtr1‐KO mice and controls (Figure 3A,B), indicating enhanced osteoclastogenesis in the absence of both genes in osteoblasts. These findings suggest that Ift20 and Wwtr1 deficiency not only impairs bone formation but also promotes bone resorption. Osteoprotegerin (OPG) is a competitive inhibitor for receptor activator of nuclear factor‐κB ligand (RANKL), thereby preventing RANKL from binding to its receptor RANK on osteoclast precursors and attenuating osteoclast differentiation [35, 36, 37, 38]. The ratio of RANKL/OPG is also reported to be an important regulator of bone homeostasis [37, 38, 39]. To further investigate whether Ift20 and Wwtr1 deficiency in osteoblasts was the direct cause of significant bone loss, we measured the expression of RANKL and OPG in osteoblasts and their circulating levels in serum of dKO mice and controls. Interestingly, we found that OPG expression was significantly decreased, whereas the RANKL expression showed no significant change in osteoblasts (Figure 3C,D). Consistently, serum analysis demonstrated decreased OPG levels and an increased RANKL/OPG ratio in dKO mice (Figure 3E–G). Moreover, the osteoblast‐osteoclast co‐culture assays confirmed that Ift20 and Wwtr1 deficiency in osteoblasts primarily orchestrates osteoclastic bone resorption in a non‐cell autonomous manner (Figure 3H,I). Collectively, these results indicate that Ift20 and Wwtr1 maintain bone homeostasis not only by supporting osteoblast differentiation but also by restraining osteoclastogenesis through modulation of the RANKL/OPG axis.

### 2.4 Ift20 and Wwtr1 Deficiency in Osteoblasts Decreases Bone Formation and Increases BMAT Accumulation

**FIGURE 4:**
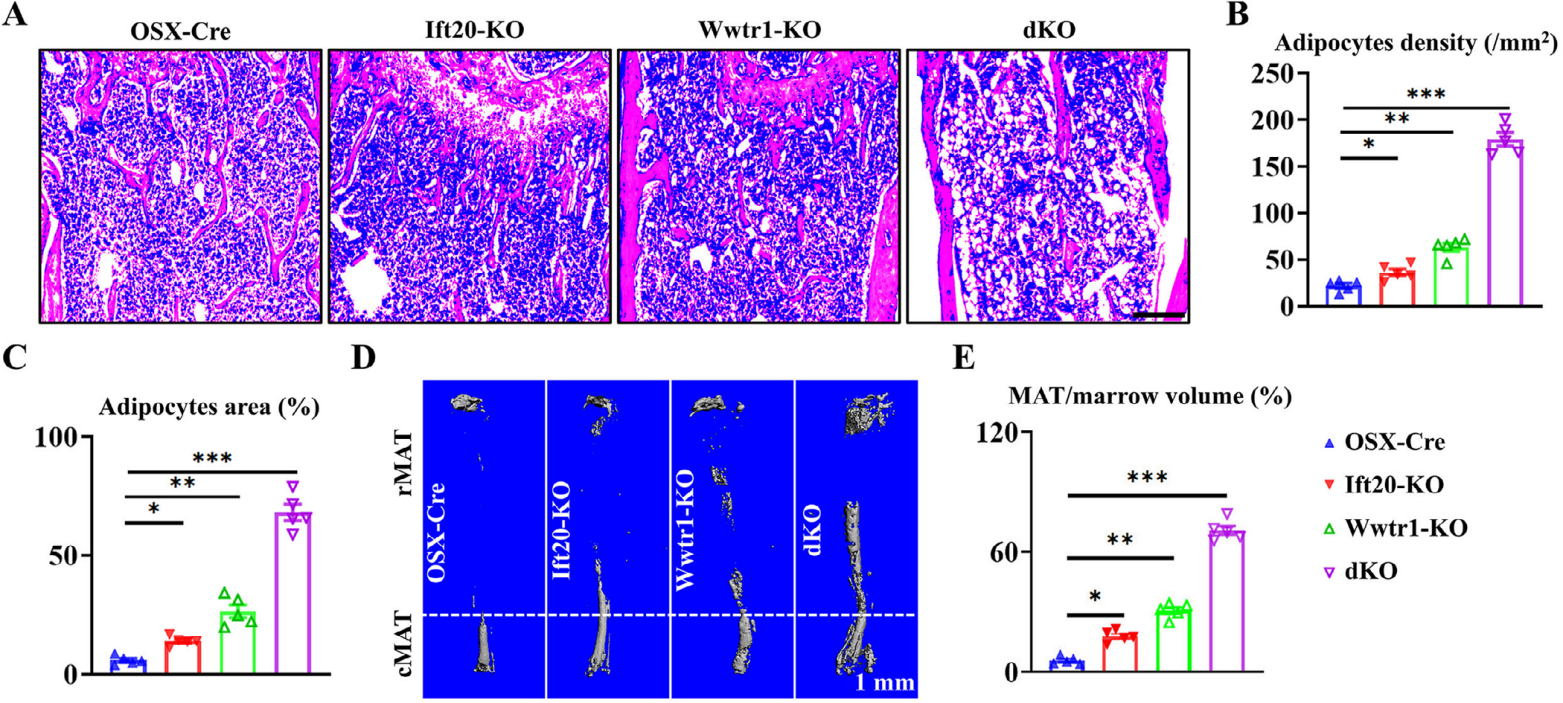
Ift20 and Wwtr1 deficiency in osteoblasts decreases bone formation and increases marrow fat accumulation. (A) Representative H&E‐stained images of femur sections from Ift20‐KO, Wwtr1‐KO, dKO mice, and age‐matched controls (OSX‐Cre mice) at 3 months. Scale bars, 100 µm. *n* = 5. (B and C) Quantifying adipocyte number and area in the distal marrow per tissue area based on H&E images. *n* = 5. (D and E) Representative µCT images of decalcified tibiae of 3‐month‐old Ift20‐KO, Wwtr1‐KO, dKO mice, and age‐matched controls after OsO_4_ staining for 2 h as indicated (D). Scale bars, 1 mm. rMAT, regulated MAT; cMAT, constitutive MAT. The qualification of MAT was identified as indicated (E). *n* = 5. Error bars: SEM. ^*^ *p* < 0.05, ^**^ *p* < 0.01, ^***^ *p* < 0.001.

Previous studies showed OP is a primary metabolic bone disease in which bone loss is accompanied by BMAT accumulation and impaired osteoclastogenesis [12, 40]. As such, we explored whether Ift20 and Wwtr1 deficiency in osteoblasts affects BMAT accumulation. Histological examination revealed a noticeable trabecular bone loss accompanied by a significant increase in the number and density of marrow adipocytes in the bone marrow milieu of dKO mice compared to the controls. In contrast, only a few adipocytes were observed in Ift20‐KO and Wwtr1‐KO mice (Figure 4A–C). These results were further confirmed by micro‐computed tomography (µCT) results, as evidenced by a significantly increased fat droplet in decalcified tibiae stained with osmium tetroxide (OsO_4_) (Figure 4D,E), suggesting that decreased bone mass may be partly due to increased adipogenesis.

### 2.5 Ift20 and Wwtr1 Synchronously Activate TβRII Signaling to Promote Osteogenesis

**FIGURE 5:**
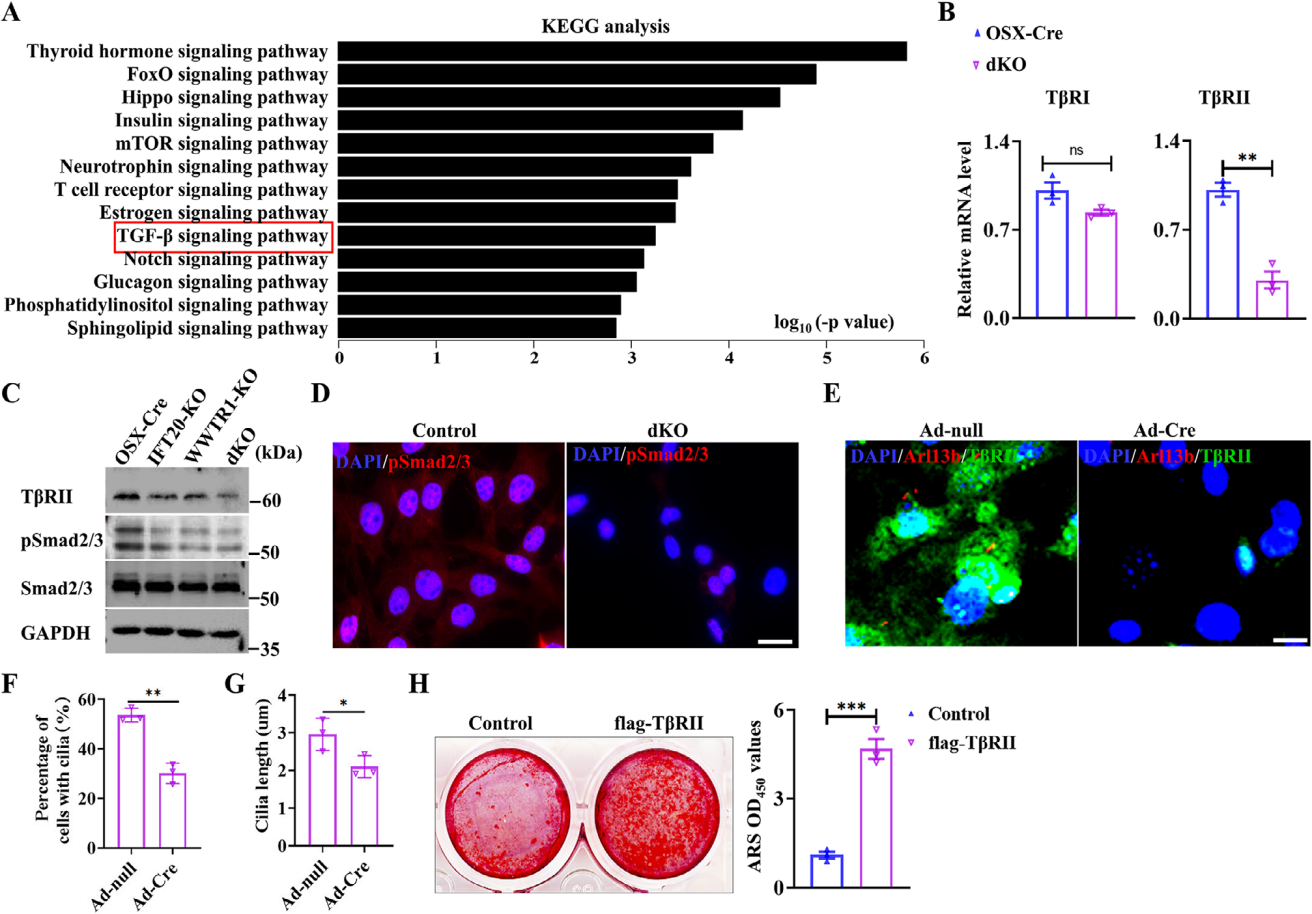
Ift20 and Wwtr1 synergistically regulate bone homeostasis by activating TGF‐β‐ TβRII signaling. (A) KEGG analysis of significant changes in genes in human osteoporosis. The red box is directed to the TGF‐β signaling pathway. (B) qRT‐PCR analysis of TβRI and TβRII in primary osteoblasts from dKO mice and age‐matched controls. *n* = 3. (C) Western blot analysis of TβRII, Smad2/3, and pSmad2/3 expression in primary osteoblasts isolated from the calvaria of Ift20‐KO, Wwtr1‐KO, dKO mice, and controls. (D) Representative fluorescence images of pSmad2/3 in primary osteoblasts as indicated. Scale bars, 20 µm. *n* = 5. (E–G) Representative co‐stained images of ciliary marker Arl13b and TβRII in primary osteoblasts from dKO mice after infection with adenovirus (Ad‐Cre) or control Ad‐null for 48 h as indicated (E). Scale bars, 10 µm. *n* = 5. The qualification of ciliated cell percentage and cilia length was identified as indicated (F and G). *n* = 3. (H) Primary osteoblasts from dKO mice were transfected with flag‐TβRII or an empty flag plasmid for 48 h. Then, the cells were induced with the osteogenic medium for 2 weeks. The alizarin red staining was performed as indicated. The intensity of alizarin red staining was measured, as shown on the right. *n* = 3. ^*^ *p* < 0.05, ^**^ *p* < 0.01, ^***^ *p* < 0.001. ns, not significant.

By analysis of the human OP database GSE35958, the Kyoto Encyclopedia of Genes and Genomes (KEGG) data revealed that the TGF‐β signaling exhibited a conserved transcriptional signature and was among the significantly enriched pathways in the analysis of downregulated genes (Figure 5A). Next, we identified the expression of TβRI and TβRII in primary osteoblasts from dKO mice and age‐matched controls. Interestingly, we found that TβRII expression was significantly decreased, but TβRI had no significant change (Figure 5B). To further investigate the potential effect of Ift20 and Wwtr1 deficiency in osteoblasts on TβRII downstream signaling, we analyzed the critical proteins downstream of the TGF‐β signaling pathway, including TβRII, pSmad2/3, and Smad2/3. Western blot analysis showed that double depletions of Ift20 and Wwtr1 in primary osteoblasts significantly reduced the expression of TβRII and pSmad2/3 compared to the single deletion (Figure 5C). Consistently, immunofluorescence staining showed a significant decrease in Smad2/3 nuclear translocation in Ift20/Wwtr1‐mutant osteoblasts (Figure 5D). These results suggested that Ift20 and Wwtr1 synchronously promote the TGF‐β signaling pathway. Our previous findings showed that Ift20 maintains cilia and cell alignment in osteoblasts [20], and governs BMSC fate through positively regulating TGF‐β‐Smad2/3‐Glut1 signaling mediated glucose metabolism [19]. By analysis of cilia marker Arl13b and TβRII expression after deletion of Ift20 and Wwtr1 in osteoblasts using immunostaining, we found that primary cilia could co‐localize with TβRII in the control group with Ad‐null infection (Figure 5E); and cilia formation was significantly inhibited along with a shortening of cilia in dKO group with adenovirus (Ad‐Cre) infection (Figure 5F,G). To further examine whether the defective osteogenesis directly results from decreased expression of TβRII caused by Ift20/Wwtr1 mutation, we overexpressed TβRII in Ift20/Wwtr1‐mutant osteoblasts and identified the osteogenic differentiation (Figure 5H and Figure S2). As expected, we found that the restoration of TβRII expression restored osteogenic differentiation in Ift20/Wwtr1‐mutant osteoblasts (Figure 5H).

### 2.6 Ift20 Inhibits the Ubiquitination and Degradation of TβRII via c‐Cbl

**FIGURE 6:**
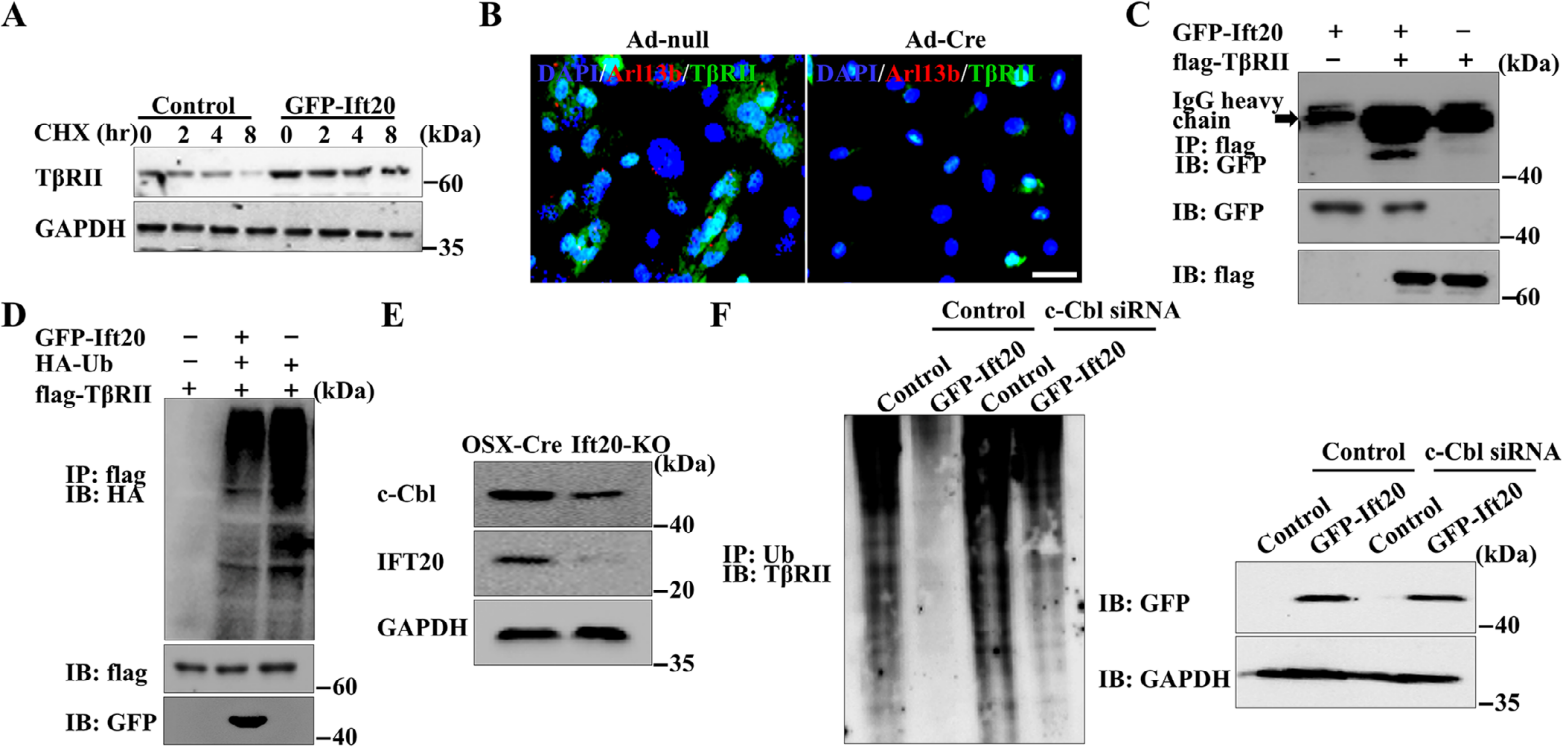
Ift20 inhibits the ubiquitination and degradation of TβRII via c‐Cbl. (A) Primary osteoblasts from the calvaria of wild‐type newborn mice were transfected with GFP‐IFT20 or GFP empty plasmid for 24 h and then treated with 50 µg/mL cycloheximide (CHX) at different times as indicated. The TβRII expression was identified by Western blot. (B) Representative co‐stained image of ciliary marker Arl13b and TβRII in osteoblasts from the Ift20^f/f^/Wwtr1^f/f^ mice with Ad‐Cre or control Ad‐null. Scale bars, 20 µm. (C) Co‐IP. After transfection of plasmid GFP‐Ift20 or/and flag‐TβRII in primary osteoblasts, the interaction of proteins Ift20 and TβRII was identified as indicated. (D) Western blot analysis of TβRII expression in primary osteoblasts co‐transfected with flag‐TβRII and/or GFP‐IFT20 and HA‐Ub for 48 h, as indicated. (E) Western blot analysis of c‐Cbl and Ift20 in primary osteoblasts from Ift20‐KO mice and controls. (F) After silence of c‐Cbl by *c‐Cbl* siRNA in Ift20‐overexpressed osteoblasts, the stability of TβRII protein was identified by Western blot.

Previous findings demonstrated that loss of Ift20 in fibroblasts promoted the autoubiquitination and proteasomal degradation of E3 ubiquitin ligase c‐Cbl [41]. Moreover, TβRII stability was inhibited by knockout of c‐Cbl in mouse embryonic fibroblasts. In contrast, overexpression of c‐Cbl improved the stability of TβRII and sensitization of cells to TGF‐β [42]. Given these findings, we speculated that Ift20 might regulate TβRII stability through c‐Cbl signaling. After treatment with the protein synthesis inhibitor cycloheximide for 48 h, we found that overexpression of Ift20 in primary osteoblasts significantly increased TβRII stability compared to the control groups (Figure 6A). Schmid et al. reported that Ift20 could interact with c‐Cbl to inhibit its autoubiquitination and degradation [41]. Given that our previous findings showed that Ift20 maintains cilia and cell alignment in osteoblasts [20], we determined whether cilia are required for TβRII stability. Our immunostaining data demonstrated that TβRII was located at the base of the cilia. Deletion of Ift20 in osteoblasts impaired cilia formation and significantly decreased TβRII expression (Figure 6B). To further characterize the function of Ift20 in TβRII stability, we overexpressed Ift20 and/or TβRII in the primary osteoblasts. Co‐immunoprecipitation (Co‐IP) data showed that Ift20 could bind to TβRII protein directly in the primary osteoblasts (Figure 6C), indicating that Ift20 may be involved in c‐Cbl‐mediated stability of TβRII protein in osteoblasts. To test whether Ift20 affects TβRII stabilization through c‐Cbl, we co‐transfected HA‐Ub with/without flag‐TβRII and GFP‐Ift20 in osteoblasts. Western blot results revealed that TβRII ubiquitination was markedly inhibited after overexpression of Ift20 (Figure 6D). Moreover, we found a significant decrease in c‐Cbl expression due to the loss of Ift20 in osteoblasts (Figure 6E). Our data further demonstrated that overexpression of Ift20 in primary osteoblasts enhanced TβRII stability by inhibiting its ubiquitination. Silencing of c‐Cbl by siRNA significantly altered the stability of TβRII; however, TβRII levels were partially restored in Ift20‐overexpressed group via decreasing ubiquitination of TβRII compared to the controls (Figure 6F), suggesting that Ift20 modulates TβRII turnover in a c‐Cbl‐dependent manner. Collectively, these data demonstrated that Ift20 preserves TβRII by inhibiting its ubiquitination and proteasomal degradation via the c‐Cbl‐mediated pathway.

### 2.7 Wwtr1 Is Required for the Endogenous Expression of TβRII

**FIGURE 7:**
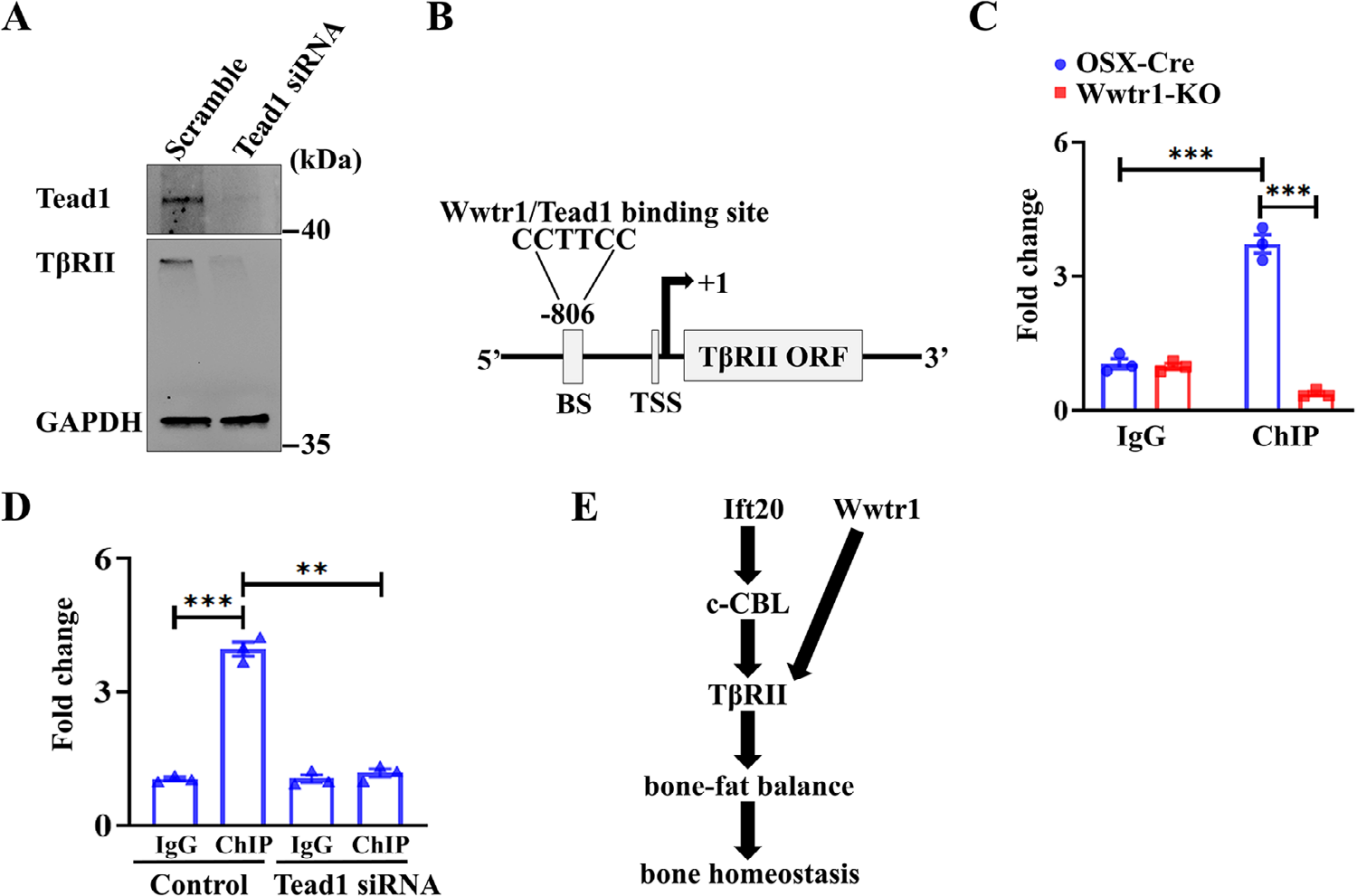
Wwtr1 is required for the endogenous expression of TβRII. (A) After silence of Tead1 using *Tead1* siRNA in osteoblasts from Wwtr1‐KO mice, the expression of TβRII was identified by Western blot. (B) Schematic binding diagram of Wwtr1/Tead1 complex in *TβRII* promoter. BS and TSS represented the binding site and transcription start site, respectively. (C) ChIP assay. Co‐occupation of Wwtr1/Tead1 complex in *TβRII* promoter in primary osteoblasts from Wwtr1‐KO mice and controls. *n* = 3. (D) ChIP assay showing reduced co‐occupancy of the WWTR1/Tead1 complex at the *TβRII* promoter following Tead1 knockdown using *Tead1* siRNA. *n* = 3. (E) The regulatory model that Ift20 and Wwtr1 synchronously regulate bone homeostasis by modulating the expression and stability of the protein TβRII. Error bars: SEM. ^**^ *p* < 0.01, ^***^ *p* < 0.001.

Emerging evidence revealed that Wwtr1 is a positive regulator of TGF‐β signaling by promoting nuclear translocation of Smad2/3 [43, 44]. Because double deletions of Ift20 and Wwtr1 pronouncedly decreased TβRII levels compared to the single deletion of Ift20 or Wwtr1, suggesting that increased TβRII level was not only caused by TβRII stability regulated by Ift20. TEA domain transcription factor 1 (Tead1) functions as an essential transcriptional co‐activator of Wwtr1 to regulate downstream regulators’ expression due to the lack of a direct DNA‐binding domain of Wwtr1 [45, 46, 47]. After silencing Tead1 by *Tead1* siRNA in primary osteoblasts from Wwtr1‐KO mice, we found that the expression of TβRII was significantly decreased compared to the control group OSX‐Cre (Figure 7A), suggesting that the complex of Wwtr1/Tead1 plays a critical role in the regulation of TβRII. To further confirm the function of the Wwtr1/Tead1 complex in the regulation of TβRII, we analyzed the binding site of the Wwtr1/Tead1 complex in the *TβRII* promoter using the Vector NTI software. As expected, we found a crucial Wwtr1/Tead1 complex binding site in the *TβRII* promoter region (Figure 7B). Therefore, we examined whether Wwtr1 regulates TβRII expression directly in primary osteoblasts. As expected, our data showed that the TβRII transcriptional activity was significantly inhibited due to loss of Wwtr1 in osteoblasts (Figure 7C). After silencing Tead1 by *Tead1* siRNA in osteoblasts from Wwtr1‐KO mice, the TβRII transcriptional activity was significantly suppressed (Figure 7D). Taken together, our data demonstrated that Ift20 and Wwtr1 synchronously regulated osteoblast differentiation via modulating TβRII expression and protein stability. Ift20 enhanced the stability of TβRII by blocking c‐Cbl‐mediated ubiquitination and degradation of TβRII; Wwtr1 increased TβRII expression by binding its promoter directly (Figure 7E).

### 2.8 Ift20 and Wwtr1 Deficiency in Osteoblasts Exacerbates Bone–Fat Imbalance in OVX‐ and HFD‐Induced OP

**FIGURE 8:**
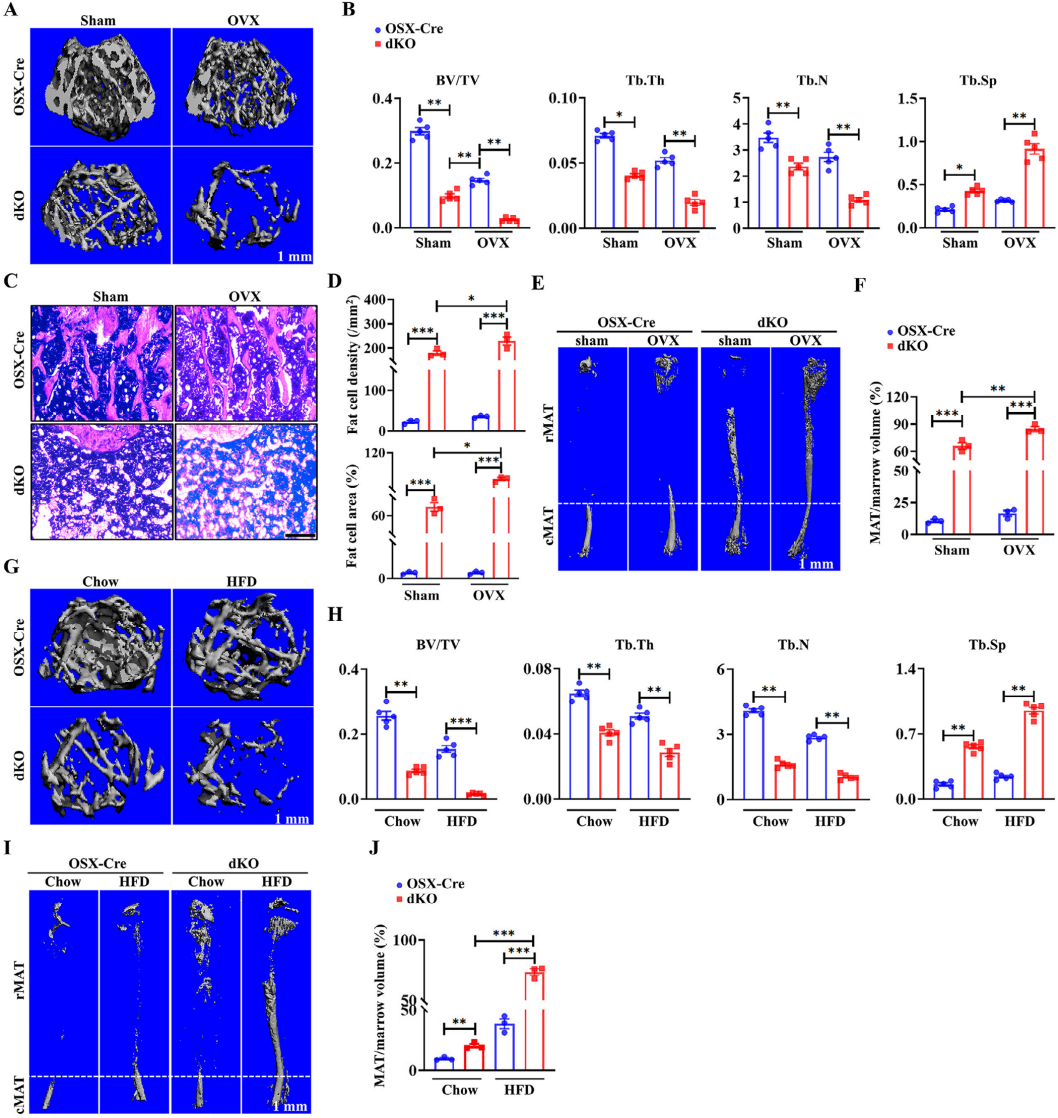
Ift20 and Wwtr1 deficiency in osteoblasts exacerbates bone–fat imbalance in OVX‐ and HFD‐induced osteoporosis. (A) Representative µCT images of trabecular bones from 3‐month‐old dKO mice and age‐matched controls after 6 weeks of ovariectomy. Scale bars, 1 mm. (B) µCT analysis of BV/TV, Tb.Th, Tb.N, and Tb.Sp in OVX samples from (A). *n* = 5. (C) Representative H&E‐stained images of femur sections from OVX samples from (A). Scale bars, 200 µm. *n* = 3. (D) Quantifying adipocyte number and area in the distal marrow per tissue area based on H&E images. (E) Representative µCT images of decalcified tibiae from 3‐month‐old dKO mice and age‐matched controls after 6 weeks of ovariectomy after OsO_4_ staining as indicated. Scale bars, 1 mm. (F) µCT analysis of BMAT in tibiae from (E). *n* = 3. (G) Representative µCT images of trabecular bones from 1‐month‐old dKO mice and age‐matched controls after feeding chow and HFD for 3 months. Scale bars, 1 mm. *n* = 5. (H) Bone parameters (BV/TV, Tb.Th, Tb.N, and Tb.Sp) were identified based on µCT analysis from (G). *n* = 5. (I and J) OsO_4_ micro‐CT staining (I) and BMAT level (J) of decalcified tibiae from 1‐month‐old dKO mice and age‐matched controls after feeding chow and HFD for 3 months by µCT analysis. Error bars: SEM. ^*^ *p* < 0.05, ^**^ *p* < 0.01, ^***^ *p* < 0.001.

To better understand the role of Ift20 and Wwtr1 in pathological bone loss, we examined the influence of Ift20 and Wwtr1 deficiency in osteoblasts on osteoporotic bone loss in mice following OVX and HFD feeding. At Week 6 following OVX, we found that dKO mice showed approximately 75% lower BV/TV compared to that in the Sham group, suggesting that Ift20 and Wwtr1 deficiency in osteoblasts exacerbated OVX‐induced OP (Figure 8A,B). The results from H&E staining of the femurs showed that the number and area of adipocytes significantly increased in dKO mice compared to those in OSX‐Cre mice after OVX (Figure 8C,D). To further confirm the effect of Ift20 and Wwtr1 deficiency in osteoblasts on BMAT formation, we conducted the µCT analysis of OsO_4_ staining of decalcified tibiae from dKO mice and controls following OVX. The dKO mice displayed a dramatic increase in the regulated MAT (rMAT) compared to the controls (Figure 8E,F). Recent studies showed that HFD is considered a crucial environmental factor in reducing bone mass and promoting BMAT expansion [40]. To further corroborate the role of Ift20 and Wwtr1 in the regulation of bone–fat balance and bone metabolism, we identified the contribution of Ift20 and Wwtr1 deficiency in osteoblasts to HFD‐induced BMAT expansion and bone loss. As expected, we found that HFD significantly reduced the bone mass of trabecular bones in dKO mice compared to age‐matched controls (Figure 8G,H). Consistently, the rMAT expansion in the bone marrow of the tibia from the dKO mice was significantly enhanced (Figure 8I,J), further supporting that Ift20 and Wwtr1 synchronously regulate bone and marrow fat homeostasis.

## 3 Discussion

Bone homeostasis is maintained through a tightly regulated balance between osteogenesis and adipogenesis within the bone marrow microenvironment. At birth, bone marrows are predominantly hematopoietic (red marrow); however, during postnatal development and aging, hematopoietic cells are progressively replaced by BMAT, resulting in conversion to yellow marrow [48]. This physiological process is further exacerbated under pathological conditions such as OP, yet the molecular mechanisms driving the pathological bone–fat shift remain incompletely understood. In this study, we identify a previously unrecognized Ift20/Wwtr1–TβRII signaling axis as a central regulator of skeletal integrity and marrow fat homeostasis.

Transcriptomic analyses of human osteoporotic bone samples revealed significant downregulation of Ift20 and Wwtr1 in osteoblast‐lineage cells. Consistent with this human data, mouse studies demonstrated that osteoblast‐specific deletion of either Ift20 or Wwtr1 impaired bone formation and increased BMAT accumulation. Notably, combined deletion in dKO mice produced a severe OP‐like phenotype characterized by profound trabecular bone loss, excessive BMAT expansion, and increased osteoclast numbers. These findings establish Ift20 and Wwtr1 as cooperative, cell‐intrinsic regulators of marrow stromal cell fate that are essential for maintaining the balance between osteogenesis and adipogenesis. While our mechanistic investigations were primarily conducted in mouse models, the concordance between human transcriptomic findings and murine genetic phenotypes supports the translational relevance of this pathway.

The importance of primary cilia and IFT proteins in skeletal biology is increasingly recognized [19, 25]. Deletion of IFT components such as Kif3a and Ift80 in osteoblasts leads to impaired bone formation and osteopenia [49, 50]. In parallel, Wwtr1 (TAZ), a transcriptional co‐activator in the Hippo signaling pathway, promotes osteogenesis while suppressing adipogenesis. Overexpression of Wwtr1 in osteoblasts increases bone mass and inhibits fat accumulation [45, 51], whereas global deletion of Wwtr1 results in ossification defects in mice [28], and impaired skeletal development in zebrafish [45]. Consistent with these observations, our results demonstrate that concurrent deletion of Ift20 and Wwtr1 in osteoblasts synergistically accelerates BMAT expansion and bone loss, underscoring their cooperative roles in maintaining postnatal bone formation and marrow adipose homeostasis.

Our in vitro mechanistic studies were performed using primary calvarial osteoblasts, a well‐established and widely used model for dissecting osteoblast differentiation and signaling pathways. We recognize that calvarial and long bones differ in their developmental origin and mode of ossification, and that OVX‐ and HFD‐induced bone loss predominantly affects trabecular bone in the appendicular skeleton. However, the in vitro defects observed in Ift20/Wwtr1‐deficient calvarial osteoblasts closely mirrored the in vivo phenotypes observed in long bones, including impaired osteoblast differentiation, increased BMAT, and enhanced osteoclastogenesis. This concordance supports the conclusion that the cilia‐dependent Ift20–Wwtr1–TβRII axis functions as a shared, cell‐intrinsic regulator of osteoblast biology across skeletal sites, although future studies directly comparing osteoblasts isolated from calvarial and long bones will be important to further define potential site‐specific differences.

Importantly, we found that Ift20–Wwtr1–TβRII axis becomes particularly critical under conditions of metabolic and hormonal stress. Both OVX and HFD, two widely used models of OP and marrow adiposity, caused exaggerated bone loss and BMAT accumulation in Ift20/Wwtr1 dKO mice compared with controls. OVX primarily induces bone loss through estrogen‐deficiency and increased osteoclast‐mediated bone resorption, whereas HFD promotes bone loss predominantly through skewing mesenchymal stem cell differentiation toward adipogenesis at the expense of osteoblast formation. The exaggerated bone loss and BMAT accumulation observed in dKO mice to both models suggest that Ift20 and Wwtr1 act as central protectors against diverse systemic stressors such as hormone and metabolic. OVX‐induced bone loss is a female‐specific model that recapitulates estrogen‐deficiency‐driven OP and therefore was appropriately performed in female mice. In contrast, HFD‐induced bone loss and metabolic dysfunction are more robust and reproducible in male mice, and the use of males for HFD studies is a widely adopted approach to minimize variability associated with estrous cycling and to ensure consistent metabolic stress [52, 53]. Importantly, the primary goal of this study was to examine how Ift20 and Wwtr1 regulate osteoblast‐intrinsic mechanisms under distinct hormonal and metabolic challenges, rather than to directly compare sex‐dependent effects. The consistent exacerbation of bone loss and marrow adiposity observed in Ift20/Wwtr1‐deficient mice across both models supports the conclusion that the Ift20–Wwtr1–TβRII axis functions as a shared regulatory mechanism under diverse pathological conditions. While these models capture key aspects of hormonal and metabolic bone loss and BMAT accumulation, future studies examining aging‐associated skeletal deterioration will be necessary to fully delineate the role of this pathway across the lifespan.

Beyond their direct effects on osteoblasts and adipocytes, our data also implicate Ift20 and Wwtr1 in the regulation of osteoclastogenesis. Previous studies, from our and other laboratories, have demonstrated that marrow adipocytes secrete RANKL, a critical osteoclastogenic cytokine, thereby linking increased BMAT to enhanced osteoclast formation and bone resorption [5, 19]. Consistently, we observed significantly elevated osteoclast numbers in dKO mice, suggesting that Ift20‐ and Wwtr1‐mediated increases in BMAT and RANKL signaling contribute to the osteoporotic phenotype. These observations reinforce the concept that the Ift20/Wwtr1 axis governs bone remodeling through coordinated effects on osteoblasts, adipocytes, and osteoclasts within the marrow niche.

Mechanistically, we identified the TGF‐β signaling, particularly through TβRII, as a key downstream effector of the Ift20/Wwtr1 axis. TGF‐β‐TβRII signaling plays a fundamental role in bone remodeling by controlling osteoblast proliferation, differentiation and coupling to osteoclast activity [54, 55, 56]. Osteoblast‐specific deletion of TβRII reduces bone mass and increases marrow adiposity [57]. Building on our previous work showing that Ift20 deficiency in BMSC impairs TGF‐β signaling and disrupts primary cilia formation [19], which is essential for osteoblast alignment and bone patterning [20], we demonstrate here that TβRII colocalizes to basal body of primary cilia, and that its stability is regulated by the E3 ubiquitin ligase c‐Cbl. This observation is consistent with prior reports demonstrating that c‐Cbl modulates TβRII stability. For example, global knockout of c‐Cbl in mice delayed bone development [58, 59], and knockout of c‐Cbl in fibroblasts decreased TβRII stability and desensitized the cells to TGF‐β stimulation [42]. In contrast, c‐Cbl overexpression stabilizes TβRII and sensitizes leukemia cells to TGF‐β [42]. This evidence strongly supported our findings that Ift20 is a crucial protein in regulating TβRII stability via c‐Cbl regulation. Besides these, Ift20 was also reported to negatively regulate the autoubiquitination and proteasomal degradation of c‐Cbl in fibroblasts [41], demonstrating the close regulation between Ift20, c‐Cbl, and TβRII. In parallel, Wwtr1 regulates TGF‐β signaling by regulating the nucleocytoplasmic shuttling of Smad2/3 [43, 44, 60]. Interestingly, we found that loss of Wwtr1 in osteoblasts inhibited TGF‐β signaling by downregulating pSmad2/3 expression. Moreover, Wwtr1 can significantly upregulate TβRII expression by directly binding its promoter. Together, these findings reveal a dual regulatory mechanism in which Ift20 controls post‐translational stability of TβRII via ciliary signaling, while Wwtr1 enhances receptor expression at the transcriptional level. This coordinated regulation establishes a previously unappreciated paradigm linking primary cilia function, transcriptional control, and receptor stability to preserve osteoblast function and suppress pathological marrow adiposity. Given the established role of primary cilia in mechanotransduction, future studies examining load‐induced bone formation and mechanical responsiveness in Ift20‐ and Wwtr1‐deficient mice will be important to determine whether this axis also participates in cilia‐dependent skeletal adaptation to mechanical stimuli.

Finally, although the present study focused on Wwtr1, it is important to note that its paralog YAP (Yes‐associated protein) is a key Hippo pathway effector that cooperates with Wwtr1 in regulating mesenchymal stem cell fate [61, 62]. Prior studies have shown that combined deletion of YAP and Wwtr1 results in severe bone loss and increased marrow adiposity, highlighting their partially redundant yet synergistic roles in skeletal homeostasis [22, 26, 63]. Whether YAP interacts with the IFT20–TβRII regulatory framework identified here remains an important question for future investigation.

Taken together, this study provides new mechanistic insights into the molecular control of postnatal bone and marrow fat development. Our findings reveal that the Ift20/Wwtr1–TβRII axis functions as a central molecular hub that integrating ciliary signaling and transcriptional regulation with TGF‐β pathway activity to maintain bone–fat homeostasis. Targeting components of this axis may offer a promising therapeutic avenue for OP and other skeletal disorders characterized by excessive BMAT accumulation.

## 4 Materials and Methods

### 4.1 Animals

OSX‐Cre, Ift20, and YAP/Wwtr1 floxed mice were purchased from the Jackson Laboratory (Bar Harbor, MA, USA). Wwtr1 floxed mice were subsequently segregated from the YAP/Wwtr1^f/f^ strain for breeding purposes. For the OVX‐induced OP model, 3‐month‐old female dKO mice and age‐matched control mice (OSX‐Cre) underwent either Sham or OVX surgery under anesthesia. Six weeks after surgery, bone mass and BMAT were assessed as previously described [19, 40]. All mice received buprenorphine (0.05–0.1 mg/kg, subcutaneous injection) for perioperative analgesia. The first dose was administered approximately 30 min prior to surgery to ensure preemptive pain control, followed by additional doses every 12 h for 48–72 h postoperatively, depending on recovery and clinical signs of discomfort. Animals were closely monitored during the postoperative period, and supportive care was provided as needed. For the HFD‐induced OP model, 1‐month‐old male dKO mice and control littermates were fed an HFD (D12492; 60% kcal from fat) for 3 months, after which the animals were euthanized for analysis. All animals were housed and maintained in accordance with protocols approved by the Institutional Animal Care and Use Committee of the University of Pennsylvania. Mice will be closely monitored for health status, and any animals exhibiting signs of pain or distress will be promptly euthanized in accordance with approved protocols.

### 4.2 Reagents and Plasmids

Antibodies against Arl13b, Smad2/3 and pSmad2/3 antibodies were obtained from Cell Signaling Technology (CST, USA). Antibodies against Tead1, TβRII, GFP, flag, Ub, GAPDH, *c‐Cbl* siRNA, and *Tead1* siRNA were ordered from Santa Cruz Biotechnology. The secondary fluorescent antibodies and H&E staining kit were ordered from Abcam. Calcein labeling, OsO_4_, and lipofectamine 3000 were purchased from Fisher Scientific. Dexamethasone, L‐ascorbic acid, and β‐glycerophosphate were ordered from Sigma. Plasmids pcDNA3.1‐flag and pcDNA3.1‐flag‐TβRII, GFP‐Ift20, and HA‐Ub were obtained from Addgene.

### 4.3 Cell Culture

Primary osteoblasts were isolated from the neonatal calvaria of Ift20‐KO, Wwtr1‐KO, dKO mice, and controls, as described before [49] and used as a well‐established mechanistic model for osteoblast differentiation and signaling analysis, whereas the functional consequences of Ift20 and Wwtr1 deletion on bone mass and marrow adiposity were evaluated in vivo in long bones. Briefly, the fresh calvaria from these mice above were cut into pieces and subjected to an enzyme solution containing 1 mg/mL collagenase Type I and collagenase Type II, collected, and cultured in α‐Minimum Essential Medium (α‐MEM; Gibco, USA) containing 1× Pen‐Strep solution (Fisher Scientific, USA) and 10% fetal bovine serum (FBS; Gibco, USA) and at 37°C with 5% humidified CO_2_. For co‐culture experiments, we performed as we previously reported [64]. Briefly, osteoblasts from wild‐type mice were seeded into a 48‐well plate (1 × 10^5^ cells/well) with α‐MEM medium. Bone marrow monocytes (BMMs) were isolated from the bone marrow of 3‐month‐old dKO mice and controls. After culturing for 24 h, the non‐adherent BMMs were collected and cultured with the osteoblasts (1 × 10^7^ cells/well) at present of 1 µM PGE2 and 10 nM 1,25‐dihydroxyvitamin D_3_. After incubation for 7 days, the cells were fixed, washed two times with PBS, and then stained with the TRAP staining kit as we previously described [19, 64].

### 4.4 ALP Activity and Osteogenic Differentiation

The primary osteoblasts from calvaria of dKO mice and age‐matched controls were cultured in osteogenic medium (α‐MEM supplemented with 100 nM dexamethasone, 50 µg/mL L‐ascorbic acid, and 5 mM β‐glycerophosphate). After induction for 5 days by osteogenic medium, the cells were harvested and incubated with the assay buffer (100 mM glycine [pH 10.5], 1 mM MgCl_2_, and 50 mM p‐nitrophenyl phosphate solution) for 15 min at 37°C. Then, the reaction was stopped by stop solution (0.1 N NaOH) and measured at OD_405_ nm by a microplate reader. ALP activity was normalized to total protein content for each sample, as previously reported [19].

For osteogenic differentiation, primary osteoblasts from Ift20‐KO, Wwtr1‐KO, dKO mice, and age‐matched controls were induced with osteogenic medium for 2 weeks. Cells were then fixed for 2 min with 4% paraformaldehyde at room temperature, stained with ARS, and analyzed following established protocols [19, 49].

### 4.5 qRT‐PCR and ChIP‐qPCR

The total RNA was extracted from primary osteoblasts using TRIzol reagent (TaKaRa, Japan). The cDNA was reverse transcribed using PrimeScript RT Kit (TaKaRa, Japan). Then, the qRT‐PCR was carried out with the SYBR Green mixture. For ChIP‐qPCR, the experiments were carried out using the Imprint Chromatin Immunoprecipitation Kit (Sigma, USA), as we previously reported [45]. The primers in this study were listed in Table S1.

### 4.6 Western Blot

The primary osteoblasts as indicated lysed with modified RIPA buffer (Thermo Fisher Scientific, USA) were harvested and subjected to SDS‐PAGE gels (Bio‐Rad, USA). After PVDF membrane transference (Millipore, USA), the membranes were immunoblotted with the primary antibodies overnight at 4°C, washed by three times with TBST (0.1% Tween‐20 in Tris‐buffered Saline), incubated with HRP‐conjugated secondary antibody for 1 h at room temperature, and analyzed by ECL solution (Thermo Fisher, USA), as we previously described [45, 64].

### 4.7 Immunofluorescence

The primary osteoblasts from the calvaria of dKO mice and controls were seeded on glass coverslips. After culturing for 48 h, the primary osteoblasts were fixed with 4% PFA and washed with 0.1% Triton X‐100 in TBST three times. The cells were blocked with 1% bovine serum albumin (BSA) for 1 h at room temperature and incubated with pSmad2/3 antibody (1:200 dilution) overnight at 4°C. After washing three times with TBST, the cells were incubated with fluorescent antibody (1:1000 dilution) and DAPI for 1 h at room temperature. Then, the cells were washed three times with TBST and visualized under a fluorescence microscope, as we previously reported [19, 45].

### 4.8 Calcein Labeling

Calcein (20 mg/kg) was administered to 3‐month‐old Ift20‐KO, Wwtr1‐KO, dKO mice, and age‐matched controls on Days 2 and 5 before sacrifice through intraperitoneal injections. After sacrifice, the tibiae from these mice above were collected and fixed with 4% PFA solution at 4°C. After washing three times, the tibiae were infiltrated with 10% potassium hydroxide solution for 3 days at 4°C. And then, the tibiae were gradually dehydrated by 70%, 80%, 95%, and 100% ethanol and xylene. Finally, the tibiae were embedded in the paraffin, sectioned, and observed by the fluorescence microscope. The Leica microanalysis system analyzed MAR and BFR, as we previously reported [19, 45].

### 4.9 Histology

Briefly, the femurs from Ift20‐KO, Wwtr1‐KO, dKO mice, and age‐matched controls were harvested and fixed in 4% PFA solution overnight at 4°C. After decalcification with 10% ethylenediaminetetraacetic acid (EDTA) in PBS (pH 7.4) for 1 month, the femurs were embedded in paraffin and then sectioned. H&E and TRAP staining were carried out using the H&E staining kit (Abcam, USA) and TRAP staining kit (Sigma, USA), respectively, as we previously reported [19, 45, 65].

TRAP staining solution was prepared according to the manufacturer's instructions. Tissue sections were incubated with the staining solution at 37°C for 30–60 min, protected from light. After staining, sections were rinsed with distilled water and counterstained lightly with hematoxylin. TRAP‐positive multinucleated cells (≥ 3 nuclei) along the trabecular bone surface were identified as osteoclasts and quantified under a light microscope. For in vitro assays, primary bone marrow‐derived osteoclast precursors were cultured on 96‐well plates in α‐MEM containing 10% FBS, M‐CSF (30 ng/mL), and RANKL (50 ng/mL) for 5–7 days. Cells were then fixed in 4% paraformaldehyde for 10 min and stained with TRAP solution at 37°C for 30 min. TRAP‐positive multinucleated cells were counted in multiple fields per well.

### 4.10 Radiographic Analysis

µCT analysis of the femurs from 3‐month‐old Ift20‐KO, Wwtr1‐KO, dKO, OVX‐ and HFD‐induced mice, and age‐matched controls was performed and analyzed by a high‐resolution µCT system, as we described previously [45, 64, 65]. For OsO_4_ MAT analysis, the whole intact tibiae of dKO mice, OVX‐ and HFD‐induced mice, and age‐matched controls were dissected, fixed in 4% PFA solution for 48 h, and decalcified in 10% EDTA for 1 month. Then, the tibiae from these mice were treated with 2% OsO_4_ solution for 2 h at room temperature. After washing for 2 days with ddH_2_O, the bones were analyzed by a high‐resolution µCT system. Quantification of fat volume, density, and distribution throughout the marrow was registered to the low contrast decalcified bone, as we previously described [19, 45].

### 4.11 Three‐Point Bending Assay

The femurs were isolated from 3‐month‐old Ift20‐KO, Wwtr1‐KO, dKO mice, and age‐matched controls (OSX‐Cre). The collected samples were subjected to three‐point bending by standard procedures at the Penn Center for Musculoskeletal Disorders (PCMD) Biomechanics Core, as we reported before [20].

### 4.12 Statistical Analysis

Publicly available transcriptomic data from OP patients (GSE35958) [30] were analyzed using R. Experimental data from this study were analyzed using GraphPad Prism 9. All experiments were performed with at least three independent biological replicates unless otherwise indicated, and technical replicates were included where appropriate. Data are presented as mean ± SEM. Normality of data distribution was assessed using the Shapiro–Wilk test. Comparisons between two groups were performed using the unpaired Student's *t*‐test. For experiments involving more than two groups or multiple factors, two‐way ANOVA was applied, followed by Tukey's or Sidak's post hoc test, as appropriate. Statistical significance was defined as *p* < 0.05.

## Author Contributions

Shuying Yang, Yi‐Ping Li, and Yang Li designed the study. Yang Li, Shuting Yang, Yang Yang, and Huijuan Chang performed experiments, analyzed data, and maintained the mice. Yang Li, Yi‐Ping Li, and Shuying Yang wrote and edited the manuscript. All authors have read and approved the final manuscript.

## Funding

This work was supported by the National Institute of Arthritis and Musculoskeletal and Skin Diseases (NIAMS, AR084491 and AR080895) and the DOD (W81XWH2110508 and W81XWH2210542), awarded to Shuying Yang. Micro‐CT Core at the University of Pennsylvania also supported this work (P30, AR069619).

## Ethics Statement

All mouse experiments (protocol #806004) were conducted in accordance with the guidelines of the Institutional Animal Care and Use Committee at the University of Pennsylvania.

## Conflicts of Interest

The authors declare no conflicts of interest.

## Supporting information




**Fig. S1**: Ift20 and Wwtr1 expression in osteoblasts. (A,B) mRNA levels of Ift20 and Wwtr1 in osteoblasts in the mice as indicated. *p < 0.05, ***p0.001.
**Fig. S2**: TβRII expression in the osteoblasts. TβRII expression was identified by western blot after transfection of the plasmid flag‐TβRII for 48 hours in the primary osteoblasts from dKO mice and controls (OSX‐Cre mice).
Supplementary primer information, Table S1.

## Data Availability

The data supporting the results of this study are available from the corresponding author upon reasonable request.
