# Supplementary material for: Ciliary Ift20 and Wwtr1 Coordinate Transforming Growth Factor‐β Receptor II Signaling to Maintain Bone–Fat Balance in Skeletal Homeostasis
Source: MedComm (2020). 2026 Sep 20;7(10):e70916. doi: 10.1002/mco2.70916 (PMC13591022; doi:10.1002/mco2.70916)
Supplement: Supplementary file 1 [file MCO2-7-e70916-s001.docx]

**Ciliary Ift20 and WWTR1 coordinate transforming growth factor-β receptor II signaling to maintain bone-fat balance in skeletal homeostasis**

Yang Li^1^^,^^2^, Yang Yang^1^, Shuting Yang^1^, Huijuan Chang^2^, Yi-Ping Li^3^, Shuying Yang^1,4,5^*

^1^Department of Basic & Translational Sciences, School of Dental Medicine, University of Pennsylvania, Philadelphia, PA 19104, USA.

^2^Department of Psychiatry and Behavioral Sciences, School of Medicine, Johns Hopkins University, Baltimore, MD 21287, USA.

^3^Division in Cellular and Molecular Medicine, Department of Pathology and Laboratory Medicine, Tulane University, New Orleans, LA 70112, USA.

^4^ Department of Periodontics, School of Dental Medicine, University of Pennsylvania, Philadelphia, PA 19104, USA.

^5^The Penn Center for Musculoskeletal Disorders, School of Medicine, University of Pennsylvania, Philadelphia, PA 19104, USA.

*Correspondence: [shuyingy@upenn.edu](mailto:shuyingy@upenn.edu)

**Fig. S1. Ift20 and Wwtr1 expression in** **osteoblasts.** (**A,B)** mRNA levels of Ift20 and Wwtr1 in osteoblasts in the mice as indicated. **P*<0.05, ****P*<0.001.

**Fig. S2.** **TβRII expression in the osteoblasts.** TβRII expression was identified by western blot after transfection of the plasmid flag-TβRII for 48 hours in the primary osteoblasts from dKO mice and controls (OSX-Cre mice).

**Fig. S1**

**Fig. S2**

**Supplementary primer information, Table S1**

| **Gene** | **sequence （5'-3'）** | **Gene** | **sequence （5'-3'）** |
| --- | --- | --- | --- |
| actin-F | AACGGCTCTGGCCTGGTGAA | Runx2 | GCCGGGAATGATGAGAACTA |
| actin-R | GCCTCATCACCCACGTAGGA | Runx2 | GGACCGTCCACTGTCACTTT |
| OCN-F | CTTGGTGCACACCTAGCAGA | OSX-F | GGAGGCACAAAGAAGCCATACGC |
| OCN-R | ACCTTATTGCCCTCCTGCTT | OSX-R | TGCAGGAGAGAGGAGTCCATTG |
| ChIP- TβRII-F | GCAACACCCTGAAAATATCA |  |  |
| ChIP- TβRII -R | TAATGAGCACCTGGGTAGAG |  |  |
